# Hybrid thoracoscopic surgical and transvenous catheter ablation versus transvenous catheter ablation in persistent and longstanding persistent atrial fibrillation (HARTCAP-AF): study protocol for a randomized trial

**DOI:** 10.1186/s13063-019-3365-9

**Published:** 2019-06-20

**Authors:** Mindy Vroomen, Mark La Meir, Bart Maesen, Justin G. L. Luermans, Kevin Vernooy, Brigitte Essers, Bianca T. A. de Greef, Jos G. Maessen, Harry J. Crijns, Laurent Pison

**Affiliations:** 1 Department of Cardiology, Maastricht University Medical Centre, PO Box 5800, Maastricht, The Netherlands; 2 Department of Cardiothoracic Surgery, Maastricht University Medical Center, Maastricht, The Netherlands; 3 Cardiovascular Research Institute Maastricht, Maastricht, the Netherlands; 4 Department of Cardiac Surgery, UZ Brussel, Brussels, Belgium; 5 Department of Clinical Epidemiology and Medical Technology Assessment, Maastricht, the Netherlands

**Keywords:** Atrial fibrillation, Persistent, Longstanding persistent, Hybrid ablation, Catheter ablation

## Abstract

**Background:**

Success rates with conventional transvenous endocardial pulmonary vein isolation in patients with persistent and longstanding persistent atrial fibrillation (AF) are variable due to advanced electrical and structural remodeling of the atria. As a consequence, more extensive endocardial lesions, minimally invasive thoracoscopic surgical techniques, and hybrid ablation (combining thoracoscopic epicardial surgical and endocardial catheter ablation) have been developed.

**Hypothesis:**

The HARTCAP-AF trial hypothesizes that hybrid AF ablation is more effective than (repeated) transvenous endocardial catheter ablation in (longstanding) persistent AF, without increasing the number of associated major adverse events.

**Methods:**

This randomized controlled trial will include 40 patients with persistent or longstanding persistent AF who will be 1:1 randomized to either hybrid ablation or (repeated) catheter ablation. The procedures and follow-up are conducted according to the guidelines. The primary effectiveness endpoint is freedom from any supraventricular arrhythmia lasting longer than 5 min without the use of Vaughan-Williams class I or III antiarrhythmic drugs through 12 months of follow-up after the last procedure. In the catheter ablation arm, a second procedure planned within 6 months after the index procedure is allowed for obtaining the primary endpoint. Additionally, adverse events, cost-effectiveness, and quality of life data will be recorded.

**Trial registration:**

ClinicalTrials.gov, NCT02441738. Registered on 12 May 2015.

**Electronic supplementary material:**

The online version of this article (10.1186/s13063-019-3365-9) contains supplementary material, which is available to authorized users.

## Background

Transvenous endocardial catheter ablation for pulmonary vein isolation (PVI) is the standard treatment option for patients with symptomatic paroxysmal atrial fibrillation (AF). Unfortunately, single procedural results of this technique in (longstanding) persistent AF are less convincing [1–5]. Due to advanced electrical and structural remodeling in these patients with nonparoxysmal AF, a more extensive lesion set might be required to achieve rhythm control. Multiple techniques have been introduced, including ablation of complex fractionated atrial electrograms (CFAE) [4–6], the application of linear lesions [4, 7–9], rotor ablation [10], or a combination of techniques in the so-called stepwise approach [11–15]. However, reported results are highly variable. One of the major limitations seems to be the availability of percutaneous devices able to reliably create transmural lesions [16].

While surgical techniques have proven to be more efficacious in overcoming this limitation [17], approaches such as the Cox-Maze procedure are considered too invasive and complex and therefore have never been widely adopted. This has led to the development of minimally invasive thoracoscopic surgical techniques and hybrid ablation. In hybrid ablation, consisting of a combination of thoracoscopic epicardial surgical and endocardial catheter ablation, surgeons and electrophysiologists combine their expertise to maximize success rates and minimize procedural complications [18]. However, the question of which lesion sets should be used in patients with nonparoxysmal AF remains unanswered. The HRS/EHRA/ECAS expert consensus statement [19] and the guidelines of the Society of Thoracic Surgeons [20] recommend considering additional ablation lesions besides PVI, but do not specify which strategy to use due to a lack of evidence. Therefore, research to identify the most appropriate lesion set and ablation technique is critical to improve results in these patients.

Currently, there is lack of clinical data comparing different ablation strategies in the treatment of nonparoxysmal AF. It is the aim of the present study to compare the safety, efficacy, and cost-effectiveness of hybrid ablation (performed in one stage) versus transvenous endocardial catheter ablation (allowing repeated ablation procedures).

## Methods

### Design and hypothesis

This is a prospective, randomized trial conducted at the Maastricht University Medical Centre, Maastricht, the Netherlands. The study objective is to compare the efficacy and safety of two interventional approaches for symptomatic, drug refractory persistent or longstanding persistent AF. A total of 40 eligible patients will be randomized (in a 1:1 ratio) to the following treatment arms: 1) a hybrid ablation arm (*n* = 20 patients), a one-stage hybrid procedure consisting of surgical thoracoscopic epicardial ablation combined with endocardial catheter ablation; or 2) a catheter ablation arm (*n* = 20 patients), consisting of conventional endocardial catheter ablation, followed by repeated catheter ablation if clinically indicated and decided within 6 months after the initial procedure.

The primary and secondary effectiveness endpoints are shown in Table 1. A blanking period of 3 months is applied. A second ablation after the hybrid ablation and a decision for redo-ablation beyond 180 days after the index catheter ablation will be considered a failure. A third catheter ablation at any time will also be considered a failure in the catheter arm. While the current guidelines define failure as a recurrence of > 30 s, failure in the primary endpoint of this study is defined as a recurrence of > 5 min [19, 21]. This allows us to reliably evaluate various Holter examinations and this reflects a more clinical endpoint compared with > 30 s, since it has been shown that the presence of AF episodes >30 s do not predict clinically meaningful AF, and that quality of life is mainly negatively influenced in patients with a high symptomatic AF burden [22, 23]. The primary and secondary safety endpoints are shown in Table 2.

**Table 1:**
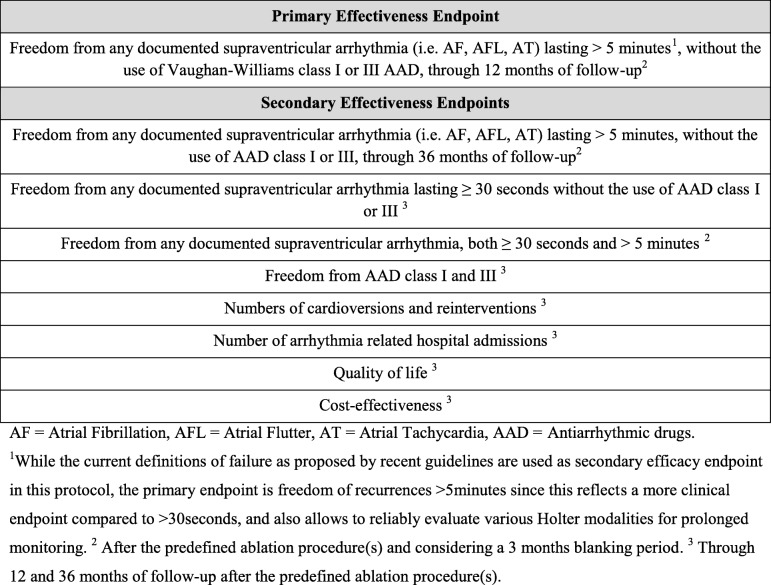
Effectiveness endpoints

**Table 2:**
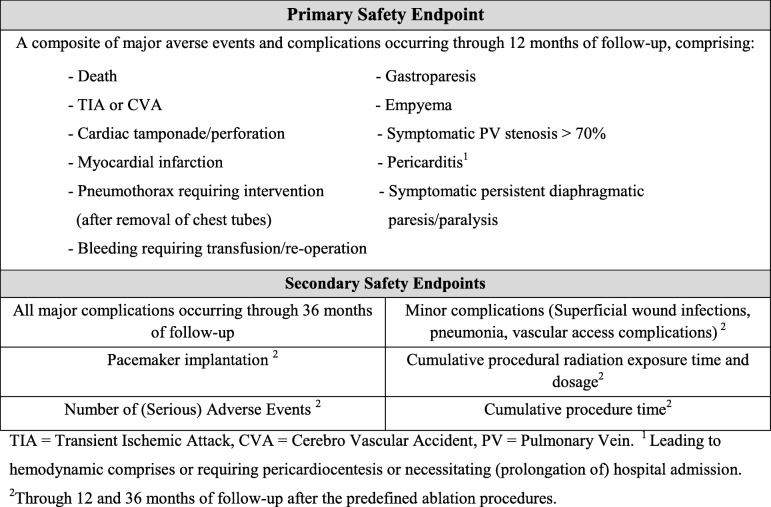
Effectiveness endpoints

This study hypothesizes that a one-stage hybrid ablation will have greater clinical effectiveness during long-term follow-up compared with (repeated) endocardial catheter ablation in patients with (longstanding) persistent AF, without increasing the number of associated major adverse events.

### Inclusion criteria

All patients (male and female, aged > 18 years, mentally able and willing to give informed consent) with symptomatic persistent or longstanding persistent AF refractory or intolerant to at least one Vaughan-Williams class I or III antiarrhythmic drug (AAD) who are able and willing to receive all of the study-related procedures are eligible for enrolment in this trial.

### Exclusion criteria

Potential subjects will be excluded from the study if any of the conditions shown in Table 3 apply.

**Table 3:**
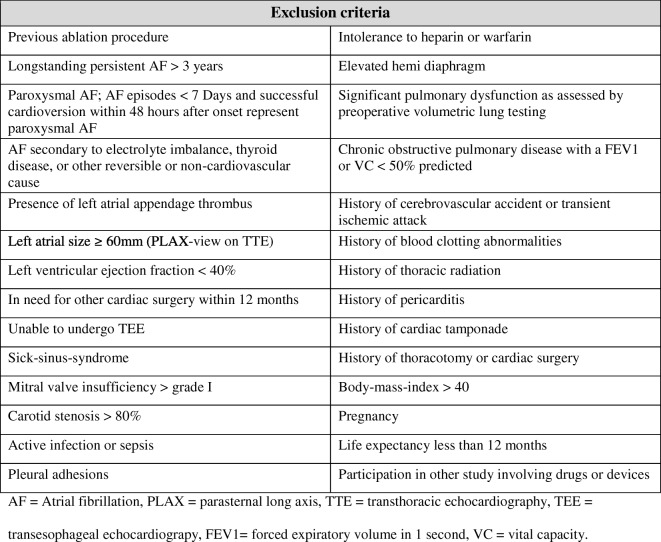
Exclusion criteria

### Randomization

Randomization will be performed directly after written informed consent is obtained by the Clinical Trial Center Maastricht using the ALEA software program (FormsVision BV, Abcoude, the Netherlands). This software is programmed to equally distribute the number of patients over the two groups so that an even number of patients are treated with either catheter or hybrid ablation. The randomization is block designed and restricted (stratified); the type of AF (persistent or longstanding AF) will be taken into account to ensure a good balance of patient characteristics in both groups. Using this randomization method, allocation concealment is guaranteed. For every patient that withdraws their consent before the ablation is performed, an additional patient will be randomized.

### Pre-procedural investigations

Pre-procedural investigations include physical examination, 12-lead electrocardiogram (ECG), blood analysis (e.g., hemoglobin, C-reactive protein, creatinine), transthoracic echocardiography, a lung function test, and a standard computed tomography (CT) scan. Patients older than 60 years and patients with an elevated cardiovascular risk profile (i.e., family history of cardiovascular disease, elevated body mass index, diabetes, dyslipidemia, hypertension, or smoking) will undergo a cardiac CT scan or coronary angiogram. In addition, quality of life (using the EQ-5D-5 L questionnaire) is assessed at baseline.

### Intervention: transvenous catheter ablation

**Fig. 1:**
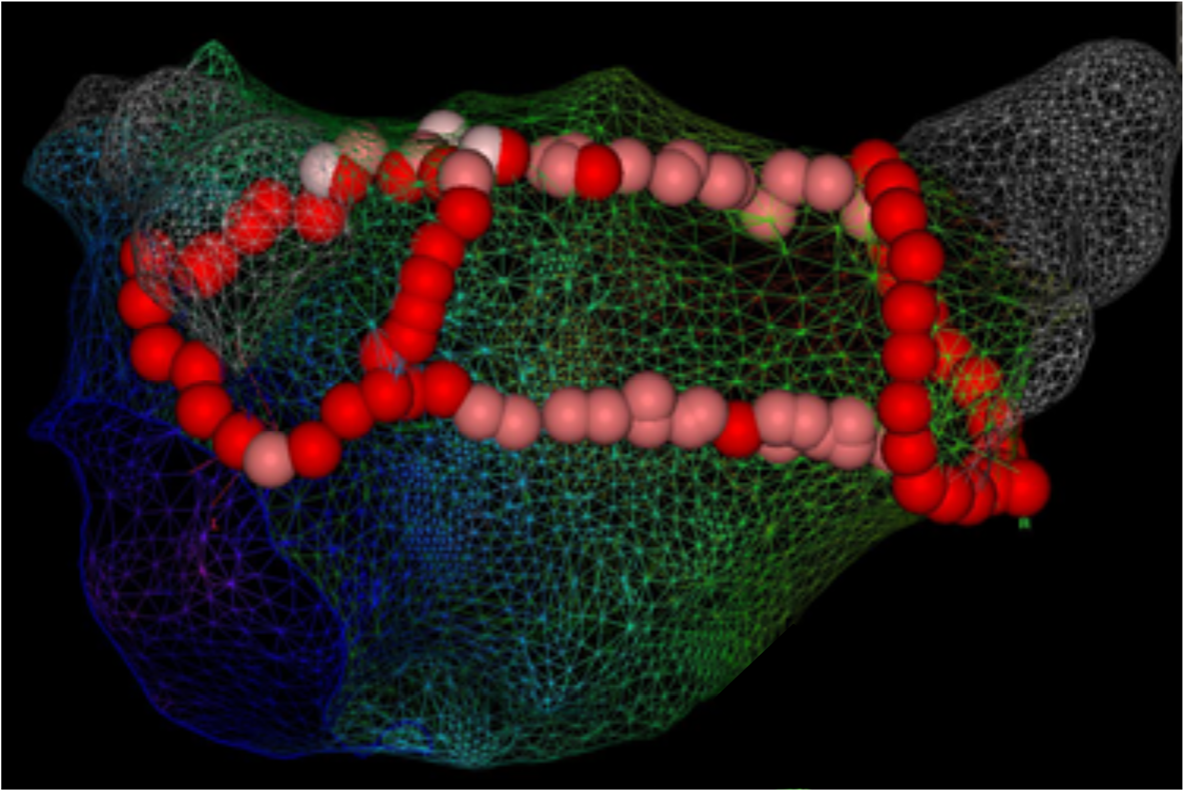
Box lesion in the setting of catheter ablation. Carto image showing a view of the posterior wall of the left atrium after wide area circumferential ablation of the pulmonary veins, and a connecting roof and inferior line (pink and red dots)

This ablation procedure is conducted while the patient is under sedation or general anesthesia. At the beginning of the procedure, a transesophageal echo is performed to confirm the absence of intracardiac thrombi. A His bundle (St. Jude Medical, St. Paul, Minnesota) and coronary sinus catheter (Medtronic, Minneapolis, Minnesota) are placed under fluoroscopy. Access to the left atrium is achieved via a femoral approach and double transseptal puncture. A single bolus of 100 IU/kg bodyweight of heparin is administered, followed by a heparin perfusor to maintain an activated clotting time above 300 s. An electroanatomical map of the left atrium is created using a mapping catheter (Lasso or Pentaray, Biosense Webster, Diamond Bar, California) with direct visualization on a three-dimensional mapping system (Carto, Biosense Webster). The ablation is performed using an irrigated tip contact force radiofrequency (RF) ablation catheter (ThermoCool Smarttouch, Biosense Webster). Automated lesion tagging (VisiTag, Biosense Webster) is used to mark the location of each ablation lesion. The power setting used is 25–30 W with an ablation index of 400 at the posterior wall, and 30–35 W with an ablation index of 500 at the anterior wall and other regions of the left atrium. The minimum lesion set that has to be performed is PVI and a superior and inferior connecting line (box lesion) (Fig. 1). In cases of a documented right atrial flutter (AFL), AFL induced during the ablation, or a dilated right atrium (> 25 mL/m^2^ in men, > 21 mL/m^2^ in women), a cavotricuspid isthmus (CTI) line is performed. Additional ablation lesions might be performed at the electrophysiologist’s discretion. Complete isolation (entrance and exit block) of all pulmonary veins and the box lesion, and completeness of additional ablation lines, must be electrophysiologically confirmed. If the patient is still in AF at the end of the procedure, the patient is electrically or pharmacologically cardioverted.

In the event of an eventual second study ablation, the lesions made in the initial ablation are checked for conduction gaps and re-ablated as needed. Additional lesions are added, if necessary, at the electrophysiologist’s discretion.

### Intervention: hybrid ablation

**Fig. 2:**
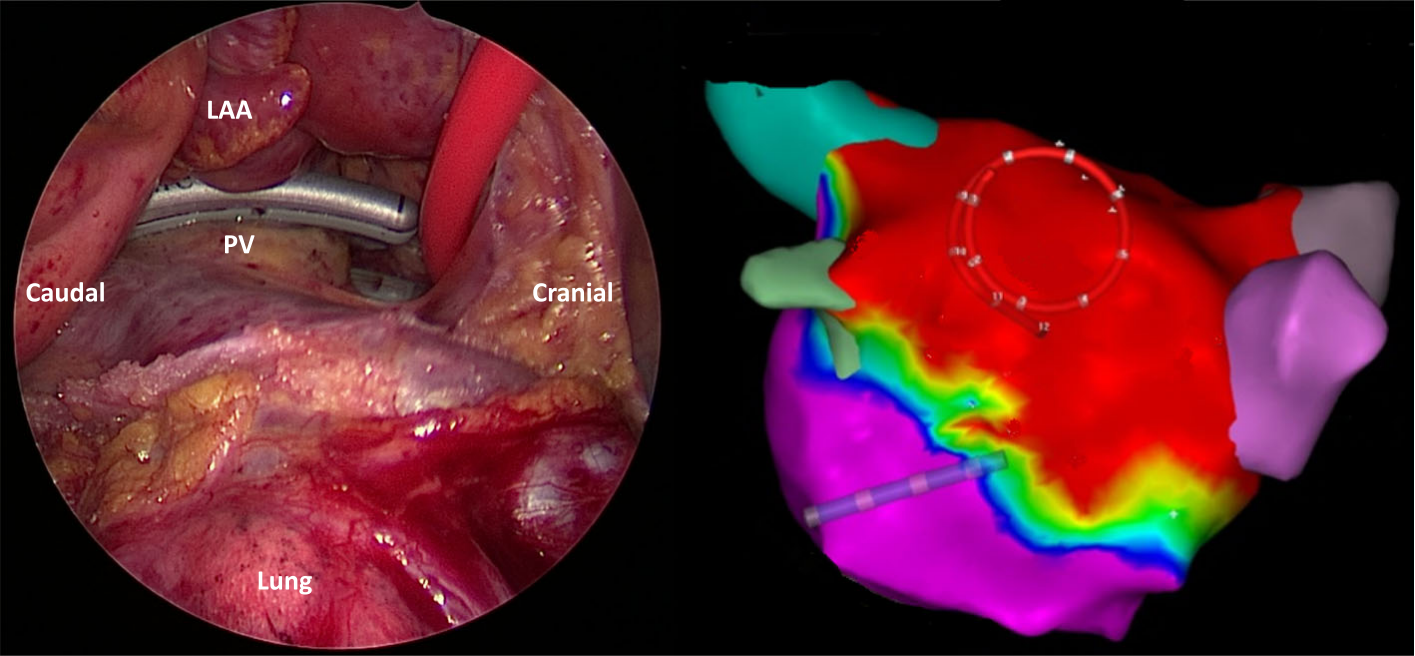
Intraoperative images of hybrid ablation. Left panel: left-sided thoracoscopic view of ablation of the left pulmonary veins using a bipolar clamp. Right panel: voltage map (Carto) after completion of the epicardial box lesion showing an isolated posterior left atrial wall. LAA left atrial appendix, PV pulmonary vein

At the beginning of this procedure a transesophageal echo is performed to confirm the absence of intracardiac thrombi. Surgical epicardial ablation is performed while the patient is under general anesthesia, with double-lumen endotracheal intubation using video-assisted thoracoscopic surgery on the beating heart. Access is gained through small incisions in the intercostal spaces. On the right side, the pericardial sac is opened parallel and ventral to the phrenic nerve. The pericardial reflections around both caval veins are dissected to provide access to the transverse and oblique pericardial sinuses. The right pulmonary veins are isolated using a bipolar RF clamp (Synergy System, AtriCure Inc., Cincinnati, OH, USA). The right-sided parts of the superior and inferior line are created using a bipolar unidirectional RF device (Coolrail, Atricure Inc., Cincinnati, OH, USA). On the left side, a similar procedure is performed. This box lesion is the epicardial lesion set in all patients regardless of their type of AF (Fig. 2). The right-sided thoracoscopic approach can be omitted if complete right pulmonary vein isolation and box lesion can be safely performed with a left-sided thoracoscopic approach only. In case of a CHA_2_DS_2_-VASc ≥ 1, closure of the left atrial appendix (LAA) using an epicardial Atriclip device (Atricure Inc., Cincinnati, OH, USA) is performed.

The epicardial surgical ablation procedure is followed by transvenous endocardial validation of exit and entrance block of the pulmonary veins and the box lesion following a single-step procedure. The same techniques used during the transvenous catheter ablation are applied to this part of the hybrid procedure. In case of incomplete lesions, conductions gaps are completed endocardially. In case of a documented right AFL, AFL induced during ablation, or a dilated right atrium (> 25 mL/m^2^ in men, > 21 mL/m^2^ in women), a CTI line is performed endocardially. Additional endocardial lesions can be added, if necessary, at the hybrid physician’s discretion. If the patient is still in AF at the end of the procedure, the patient is electrically or pharmacologically cardioverted.

### Follow-up

**Fig. 3:**
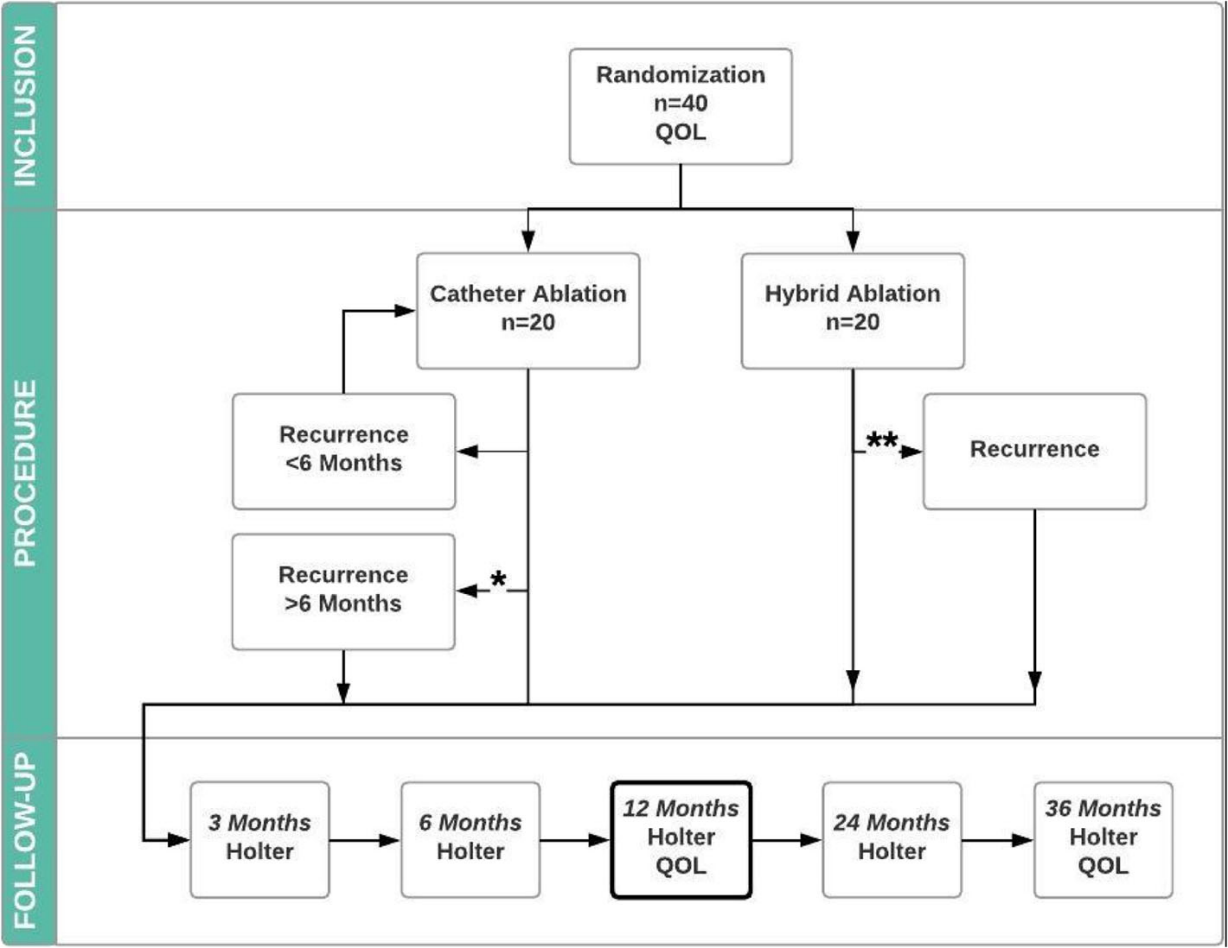
Study flow chart. *A decision for a second ablation > 6 months after the index procedure, and a third ablation at any time are considered a failure. **A second procedure is always considered a failure. The primary endpoint is set at 12 months after the predefined ablation procedure(s). QOL quality of life

**Fig. 4:**
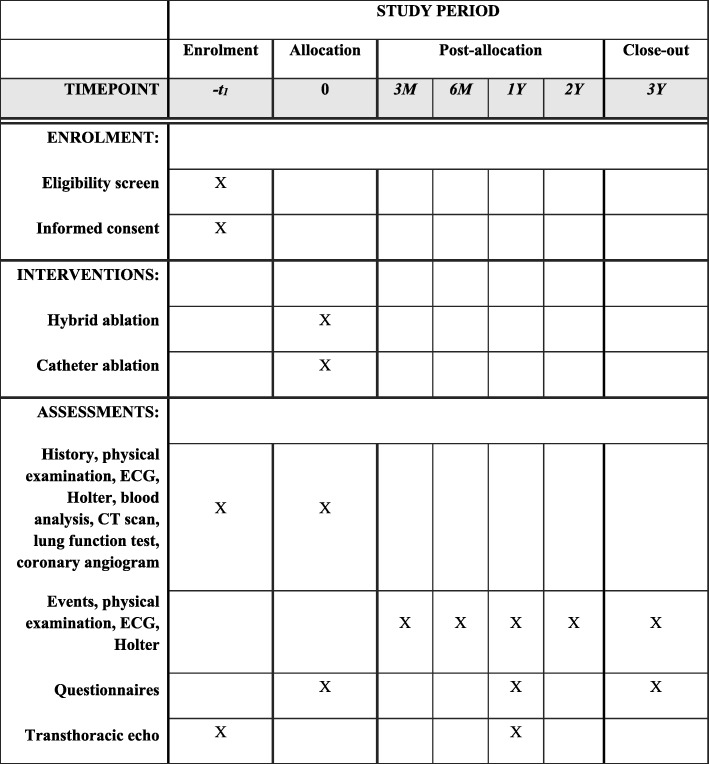
SPIRIT figure. CT computed tomography, ECG electrocardiogram

Patients are seen at the outpatient clinic 3, 6, 12, 24, and 36 months after the predefined ablation procedures. At all visits the heart rhythm will be determined according to the HRS/EHRA/ECAS expert statement [24], which includes an ECG in case of symptoms and at each visit, 24-h Holter examination at 3 and 6 months, and a 7-day Holter examination at the remaining visits. Furthermore, in case of an absence of symptoms on the ECG, Holter examinations are performed. The Holter examinations are assessed by nonblinded but independent Holter specialists. At 12 and 36 months, quality of life (using the EQ-5D-5 L questionnaire) is re-assessed (Fig. 3). The SPIRIT checklists are presented in Fig. 4 and Additional file 1.

### Cost-effectiveness

The economic evaluation will examine the cost-effectiveness of hybrid AF ablation compared with (repeated) transvenous endocardial catheter ablation from a societal perspective with a time horizon up to 36 months. The outcome measure will be the incremental costs per quality-adjusted life year (QALY) gained. To calculate the QALY, a generic health-related quality of life questionnaire, the EQ-5D-5 L, will be administered at baseline and at 12 and 36 months. The Netherlands tariff will be used to value the health states as experienced by patients [25].

All resource use related to both procedures within the hospital will be recorded. In addition, medical consumption of patients during follow-up will be recorded using the iMCQ, while productivity loss will be obtained using the iPCQ [26, 27]. For the valuation of resource use, unit prices will be derived from the Pharmacotherapeutic Compass, the Netherlands guidelines for cost calculation [28], and the hospital financial department.

### Sample size calculation

The estimated success rate (freedom from any supraventricular arrhythmia lasting > 5 min without the use of Vaughan-Williams class I or III AAD through 12 months of follow-up after the last procedure) based on literature and our own clinical experience is 35% for single catheter ablation [2, 29] and 83% for a hybrid ablation [30, 31]. With a significance level of α = 0.05, and a power of 90%, 20 patients per group need to be included.

### Statistical analysis

Data analysis on the final dataset will be performed using SPSS statistical software. The per-protocol principle will be used since patients who withdraw consent before the ablation has taken place are excluded from the analysis. Univariate analysis will be applied to both continuous (*t* test or nonparametric tests) and categorical variables (Chi-square test). Continuous variables will be reported as the mean ± standard deviation (for normally distributed variables) or median/p10–p90 (for not normally distributed variables), and categorical variables will be reported as the observed number and corresponding percentage. Depending on the number of events, univariate (*t* test or Chi-square) or multivariate analysis (logistic regression) will be used to explore the relationship between baseline covariates and post-baseline endpoints. Kaplan-Meier survival curves will be plotted. All necessary decisions with regards to power calculations for secondary endpoints are made blind. Missing data are not computed or imputed, and the participant will be excluded for the analysis which involves the missing data.

For the cost-effectiveness analysis, any missing data will be imputed using a multiple imputation approach. As most volumes of resource use follow a skewed distribution, differences between the two groups will be analyzed with bias-corrected bootstrap analysis. In addition, bootstrap analysis will also be used to quantify the uncertainty surrounding the incremental cost-effectiveness ratio. Results of this analysis will be presented in cost-effectiveness planes and acceptability curves. Sensitivity analysis will be performed to estimate, for example, the impact of parameters on the incremental cost-effectiveness ratio. Discounting will be applied for both costs (4%) and effects (1.5%).

### Ethics

Eligible candidates will be screened while considering the inclusion and exclusion criteria. Oral and written information is given to all eligible candidates. If written informed consent is obtained, the patient is enrolled in the trial and randomized to one of the treatment arms. Both the ablation strategies studied herein are currently accepted and recognized in the guidelines [19, 20, 24, 32]. Adequate clinical experience with the procedures was obtained before the present study. Full reimbursement of costs for both therapies is granted by the Dutch healthcare system. Their comparison complies with the Declaration of Helsinki for the ethical principles of medical research in human subjects. The Institutional Review Board of Maastricht University Medical Centre+ and Maastricht University (azM/UM, the Netherlands) gave full approval for the study. The study is registered in Clinicaltrials.gov under number NCT02441738. A Data Safety Monitoring Board has been formed, and its members are listed in Appendix. Adverse and serious adverse events will be reported to the Data Safety Monitoring Board and the Institutional Review Board. A clinical research monitor of the Clinical Trial Center Maastricht regularly monitors the conduct of the study.

## Discussion

The aim of this single-center, randomized study is to compare the safety and efficacy between hybrid ablation and (repeated) catheter ablation for patients with nonparoxysmal AF. The results will provide new insights into the optimal interventional treatment for patients with nonparoxysmal AF, as well as associated cost-effectiveness and quality of life.

Due to the complexity of the underlying pathology, nonparoxysmal forms of AF are challenging to treat. Endocardial PVI results in single-procedure success rates of 23–48% in this patient subgroup [1–5]. However, with a second procedure and the use of AADs, outcomes can be improved to 46–69% [1–4, 33]. While PVI is still the cornerstone of the treatment of any type of AF, the appropriate substrate modification approach for nonparoxysmal AF is controversial. While the addition of CFAE ablation to PVI improved outcome in the STAR-AF I trial (74% vs. 48%, *p* = 0.004), neither the STAR-AF II trial (59% vs. 49%, *p* = 0.15) nor a large meta-analysis of 1415 patients (29.7% vs. 31.3%, *p* = 0.17) could confirm this benefit [4–6]. Furthermore, the stepwise approach consisting of PVI followed by disconnection of caval veins, CFAE ablation, and linear ablation of the roof and mitral isthmus until sinus rhythm is restored showed inconsistent results [11–15, 34]. In addition, it has been shown that full endocardial ablation of the posterior wall, which includes the application of linear lesions, does not always improve outcomes [7]. Other techniques exist, such as focal impulse and rotor modulation and isolation of low voltage areas, but without definitive positive results. In our experience, out of a total of 70 initial RF catheter ablations, performed between 2012 and 2015, 60 patients suffered from persistent AF and 10 from longstanding persistent AF. In 54 patients, only PVI was performed (77%), while in the remaining patients AT ablation (2%), CTI line (17%), and/or CFAE ablation (4%) was added. The median follow-up period was 21 months (range 15–30 months). The single-procedure success rate was disappointing, with only 21% of patients being free of supraventricular arrhythmias without AADs, and 27% with the use of an AAD. In 31/51 recurrences, a redo-procedure was performed, of which five were His bundle ablations. The pulmonary vein reconduction rate was 70% of all pulmonary veins. After the redo-procedure, the success rate without the use of AADs improved to 43%, and with the use of an AAD to 48%. The variety of reported success rates has led to different recommendations in consensus documents and guidelines. The most recent ESC guidelines do not recommend more extensive endocardial ablation during a first ablation attempt since there is not enough evidence to justify the prolonged procedure and radiation times, and the possibly higher complication rates (class IIa) [32]. On the other hand, the 2017 HRS/EHRA/ECAS expert consensus states that posterior wall isolation using a superior and inferior line in catheter ablation of nonparoxysmal AF may be considered (class IIb) [19]. In the surgical community, consensus has been achieved that additional lesions in minimally invasive off-pump procedures for nonparoxysmal AF are recommended [20] since PVI alone does not achieve outcomes comparable to minimally invasive versions of the on-pump maze procedure (60% vs. > 80%) [35–37]. Therefore, we decided to apply the same lesion set consisting of PVI and isolation of the posterior atrial wall (a so-called box lesion) in both arms of this study.

One reason for the inconsistent results and high failure rates might be the limitations of the devices used to create transmural lesions. For example, in the STAR-AF II trial standard unipolar endocardial uniparietal catheter techniques were used, which are known to not reliably produce long-lasting transmural lesions [38]. Incomplete linear lesions can be arrhythmogenic due to the possibility for re-entry at the site of gaps or by changing conduction velocity [39]. However, even if current state of the art technology is applied, as in the recent Fire and Ice trial, the limitations of endocardial ablation in reliably creating transmurality currently still remain evident [40]. Surgical bipolar and biparietal devices are better at creating transmural lesions, and modern technologies allow for an anatomical approach using thoracoscopic visualization. However, epicardial surgical ablation is unable to create efficacious isthmus lesions and lacks the opportunity of sophisticated high-density three-dimensional mapping and consecutive endocardial touch-up of gaps. A hybrid ablation, which encompasses all of these opportunities, can lead to single-procedure success rates of more than 85% freedom from AF without AADs [18, 30, 31].

Besides efficacy in terms of rhythm control, safety also determines if an approach can be recommended. The advantages of catheter ablation are that full sedation is not needed, the duration of admission is short, and recovery is fast. The complication rates of this procedure vary from 0.2% to 4% per complication, with a cumulated complication risk of 7.7% to 13% [32, 41], which we have confirmed in our center. A hybrid ablation gives a total minor complication rate of 4% and total major complication rate of 5% [42]. In our center, we have confirmed these reported complication rates [30].

Available research has shown that patients, especially those presenting with longstanding persistent AF, might benefit from a hybrid procedure compared with surgery only [43]. Furthermore, it is known that patients with failed catheter ablation show better results after a hybrid procedure compared with a redo-catheter ablation [44]. The randomized FAST trial [17] compared surgical ablation with catheter ablation. Surgical ablation was superior to catheter ablation in terms of outcome but was accompanied by more adverse events. A retrospective study of 166 patients also showed favorable results with regard to the rhythm outcome of surgical ablation over catheter ablation, with no significant differences in complication rates [45].

Consequently, a hybrid procedure combining the strengths of an epicardial and endocardial approach should overcome some of the shortcomings of each technique and enhance the outcome in the difficult to treat patient subgroup with nonparoxysmal AF. However, catheter ablation and hybrid ablation have never been compared in a trial. This gap in the literature shows the need for this study to investigate the safety and effectiveness of catheter and hybrid ablation in a randomized setting while also addressing associated cost-effectiveness to facilitate decision making in the treatment of patients with nonparoxysmal AF.

### Trial status

At the time of submission, the trial is recruiting patients. The first procedure was performed on 30 January 2017.

### Additional file

Additional file 1: SPIRIT checklist: recommended items to address in a clinical trial protocol and related documents. (DOC 120 kb)

## Appendix

The members of the Data and Safety Monitoring Board for this trial are: Professor Dr. M.H. Prins, Statistician (The Netherlands), Chair; Professor F. Wellens, Cardiothoracic surgeon (Belgium); and Professor T. Gorgels, Cardiologist (Netherlands). The members declare that they have no conflict of interest with the sponsor of the study. A charter describing the roles and responsibilities of the independent Data and Safety Monitoring Board was made, including the timing of meetings, methods of providing information to and from the data and safety monitoring, frequency and format of meetings, statistical issues, and relationships with other committees.

## Abbreviations

AAD: Antiarrhythmic drug
AF: Atrial fibrillation
AFL: Atrial Flutter
CFAE: Complex fractionated atrial electrograms
CTI: Cavotricuspid isthmus
PVI: Pulmonary vein isolation
QALY: Quality-adjusted life year
